# Holocene El Niño–Southern Oscillation variability reflected in subtropical Australian precipitation

**DOI:** 10.1038/s41598-019-38626-3

**Published:** 2019-02-07

**Authors:** C. Barr, J. Tibby, M. J. Leng, J. J. Tyler, A. C. G. Henderson, J. T. Overpeck, G. L. Simpson, J. E. Cole, S. J. Phipps, J. C. Marshall, G. B. McGregor, Q. Hua, F. H. McRobie

**Affiliations:** 1 Department of Geography, Environment and Population, The University of Adelaide. North Terrace, Adelaide, South Australia 5005 Australia; 2 Sprigg Geobiology Centre, The University of Adelaide. North Terrace, Adelaide, South Australia 5005 Australia; 3 School of Biosciences, Sutton Bonington Campus, University of Nottingham, Leicestershire, LE12 5RD UK; 4 Stable Isotope Facility, Centre for Environmental Geochemistry, British Geological Survey, Nottingham, NG12 5GG UK; 5 Department of Earth Sciences, The University of Adelaide. North Terrace, Adelaide, South Australia 5005 Australia; 6 School of Geography, Politics and Sociology, Newcastle University, Newcastle upon Tyne, NE1 7RU UK; 7 School for Environment & Sustainability, The University of Michigan, Ann Arbor, Michigan 48109 USA; 8 Institute of the Environmental Change and Society, University of Regina, Saskatchewan, S4S 0A2 Canada; 9 Department of Earth and Environmental Science, The University of Michigan, Ann Arbor, Michigan 48109 USA; 10 Institute for Marine and Antarctic Studies, University of Tasmania, Hobart, Tasmania Australia; 11 Queensland Department of Environment and Science, Dutton Park, Queensland, 4102 Australia; 12 Australian Nuclear Science and Technology Organisation. Locked Bag 2001, Kirrawee DC, New South Wales 2232 Australia; 13 School of Mathematics and Statistics, University of Western Australia, Crawley, Western Australia 6009 Australia

## Abstract

The La Niña and El Niño phases of the El Niño-Southern Oscillation (ENSO) have major impacts on regional rainfall patterns around the globe, with substantial environmental, societal and economic implications. Long-term perspectives on ENSO behaviour, under changing background conditions, are essential to anticipating how ENSO phases may respond under future climate scenarios. Here, we derive a 7700-year, quantitative precipitation record using carbon isotope ratios from a single species of leaf preserved in lake sediments from subtropical eastern Australia. We find a generally wet (more La Niña-like) mid-Holocene that shifted towards drier and more variable climates after 3200 cal. yr BP, primarily driven by increasing frequency and strength of the El Niño phase. Climate model simulations implicate a progressive orbitally-driven weakening of the Pacific Walker Circulation as contributing to this change. At centennial scales, high rainfall characterised the Little Ice Age (~1450–1850 CE) in subtropical eastern Australia, contrasting with oceanic proxies that suggest El Niño-like conditions prevail during this period. Our data provide a new western Pacific perspective on Holocene ENSO variability and highlight the need to address ENSO reconstruction with a geographically diverse network of sites to characterise how both ENSO, and its impacts, vary in a changing climate.

## Introduction

**Figure 1:**
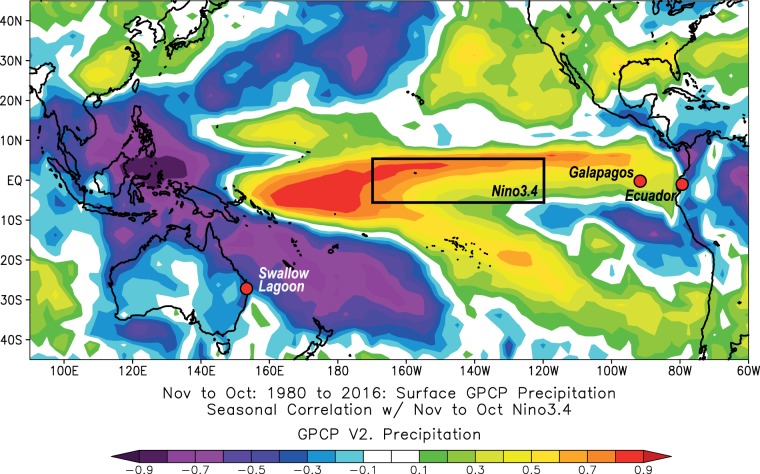
ENSO influence on surface precipitation. Spatial correlation between mean precipitation (Nov–Oct: the local hydrological year^17^) across the greater ENSO region and mean sea surface temperature for the Nino3.4 region (box) for the period 1980–2016 CE. Location of the study site and other locations mentioned in the text are illustrated.

The El Niño-Southern Oscillation (ENSO) describes variation in tropical Pacific Ocean temperatures and the resulting changes in atmospheric pressure gradients. The atmospheric changes widely propagate the effects of ENSO variability, making ENSO a major component of regional climate across much of the world^1^. The impacts of changes in regional temperature and precipitation patterns associated with El Niño and La Niña phases of ENSO have wide-ranging environmental, societal and economic consequences. The El Niño phase manifests as a warming of central and/or eastern Pacific sea surface temperature (SST) with resulting increased precipitation in northern South America and western North America (Fig. 1). Conversely, the associated cooling in the western Pacific during El Niño events is associated with drought, forest fires and reduced agricultural yield in the western tropical Pacific, including the eastern half of Australia^2^. The opposing La Niña phase is equally important as a driver of drought in the eastern Pacific and positive precipitation anomalies in the west Pacific^2^. This was most recently evident during the 2010/11 La Niña, when the volume of precipitation over land was sufficient to reduce global sea levels by 5 mm, with much of this falling on Australia^3^. This resulted in catastrophic flooding in the sub-tropics and massive carbon uptake via greening of the vast arid and semi-arid regions of the continent^4,5^.

Given these wide-ranging effects, it is essential to understand how both phases of ENSO will respond to future climate change. Reducing predictive model uncertainties requires proxy data of ENSO behaviour under different background states, as well as in response to local and extra-regional influences from all ENSO-sensitive areas^6,7^. The Holocene provides fruitful opportunities for this, with millennial-scale changes in orbital radiation forcing and centennial-scale global temperature changes, such as the Little Ice Age (~1450–1850)^8^. However, the evolution of ENSO through the Holocene remains unclear, with discrepancies between central Pacific SST proxies^9^ and eastern Pacific proxies of both precipitation^10^ and SST^11^, particularly during the mid-Holocene. Additionally, there are very few proxy ENSO records that can resolve centennial-scale trends in changing ENSO mean state. This is important as changes in the dominant phase of ENSO have been linked to solar irradiance^12^, orbital forcing^13^, average global temperatures^14^ and fresh water fluxes in the North Atlantic^7^.

We present a new ~7700-year quantitative precipitation record from subtropical eastern Australia, where La Niña and El Niño conditions are associated with positive and negative rainfall anomalies, respectively^2^ (Fig. 1). The precipitation reconstruction is derived from the carbon isotope ratio (δ^13^C) of leaves from the evergreen tree *Melaleuca quinquenervia* ((Cav.) S.T. Blake) preserved in the Holocene sediments of Swallow Lagoon on Minjerribah (North Stradbroke Island), the world’s second largest sand island. Swallow Lagoon (27°29′55″S: 153°27′17″E) is a small (0.27 ha), perched, freshwater lake that is isolated from the regional water table^15^. With no inflow or outflow streams, the balance of precipitation over evaporation determines lake level (Fig. S1) and moisture availability for the isolated stand of *M. quinquenervia* that fringes the lake (Supplementary Information). Sediments from a 370 cm core were sieved at contiguous one-centimetre resolution for *M. quinquenervia* leaf fragments, yielding 284 samples. Each datum represents the δ^13^C of all leaf fragments at that depth and is an average for the period encapsulated by that centimetre of sediment, which ranges from two to 77 years (avg. 24.4 yrs; s.d. 15.6 yrs). As such, these data do not represent El Niño or La Niña events, but represent mean conditions of individual time-slices. Age control is provided by 18 accelerator mass spectrometry ^14^C dates on short-lived terrestrial macrofossils, including *M. quinquenervia* leaves (Table S1).

Our new rainfall reconstruction builds on a well-established relationship between carbon isotope fractionation in C_3_ plant leaves and moisture availability (e.g., ref.^16^). In a novel approach, we utilise a relationship established specifically for *M. quinquenervia* using a 12-year collection of monthly litterfall samples from a nearby south-east Queensland wetland, which demonstrated a linear relationship (*r*^2^ = 0.67, *p* = 0.002) between the carbon isotope discrimination of *M. quinquenervia* leaves, relative to atmosphere, and mean annual rainfall^17^. We apply this calibration to sub-fossil *M. quinquenervia* leaf fragments from Swallow Lagoon to derive a quantitative estimate of mean annual rainfall. The linear nature of the model may skew precipitation estimates to the lower end and affect apparent variability; however, our calibration has advantages over other potential datasets as it uses location-specific climate data and is species-specific, as opposed to using modelled rainfall estimates^16^ or averaged data from all C_3_ plants at a location^18^. Comparing our results against various reconstructions from global datasets demonstrate that they consistently reconstruct higher precipitation estimates, however the patterns of change and variance, although accentuated, do not differ and our findings based on the species-specific calibration remain robust (Fig. S4).

**Figure 2:**
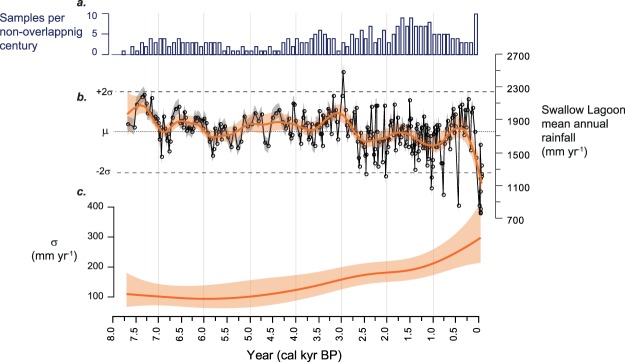
The Swallow Lagoon precipitation record. (**a**) The number of individual samples per non-overlapping century. (**b**) The Swallow Lagoon rainfall reconstruction with standard error (±88 mm; grey shading) and generalised additive location-scale model (GAM-LS: orange line with 95% confidence level shaded) illustrating significant trends in the data. Horizontal black dashed lines indicate ±2σ of the record, dotted line is the mean (1742 mm). (**c**) Standard deviation (σ) in mean annual rainfall with 95% confidence level shaded (see methods).

The inferred rainfall record from Swallow Lagoon covers the last 7700 years (Fig. 2) and displays a transition from predominantly high precipitation with low frequency variability during the mid-Holocene, to a drier climate with enhanced centennial-scale variability after ca. 3200 cal yr before present (3.2 cal kyr BP, where ‘present’ is 1950 CE). However, both non-constant sampling through time and varying numbers of years per sample could affect the variability in our record. To assess the fidelity of this shift in variability, we use a generalized additive location scale model (GAM-LS) to simultaneously estimate trends in both the mean (*μ*) and the standard deviation (σ) of the rainfall record. We find a statistically significant trend in σ. To test if this trend was influenced by sampling resolution, the estimated model was tested against a null model using 1000 simulated time series that follow the nonlinear trend estimated by our GAM-LS model but importantly with constant variance. This process demonstrates the range of trends in σ we might expect if there were no systematic change in variance. Simulation results demonstrate the estimated trend in σ is not an artefact arising from varying sampling resolution in time (Fig. S5). The combination of the fidelity of the variability in the record, the similarities between this and the general pattern of Holocene ENSO variability seen in other proxy records^11,19–21^, and the ENSO-sensitive location of the study region, provide confidence that rainfall variability in the record reflects ENSO variability through the Holocene. We therefore interpret the record in terms of mean ENSO conditions of individual time slices, as discussed above. Alternative explanations involving changes in ENSO ‘flavour’^22^ or shifting teleconnection patterns^23,24^ are not as firmly grounded in the palaeoclimate literature, although we cannot rule these out.

**Figure 3:**
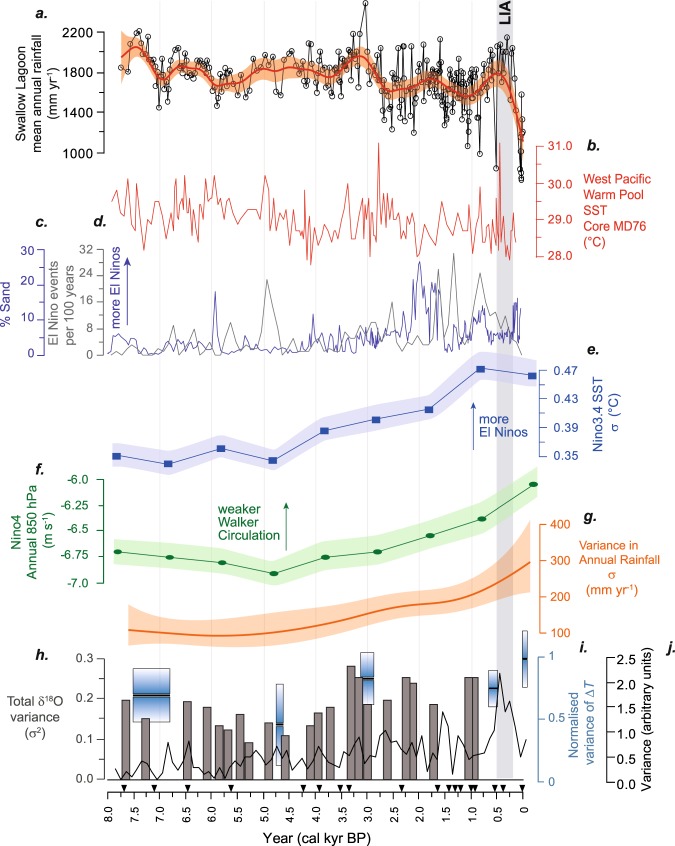
(**a**) Swallow Lagoon precipitation record (as per Fig. 2); (**b**) West Pacific warm pool SST^32^; (**c**) lake sediment sand content from El Junco Lake, Galápagos Islands^19^, and (**d**) sediment deposition at Laguna Pallcacocha, Ecuador^25^, as proxies for El Niño event frequency; (**e**) simulated amplitude of ENSO variability as reflected by Nino3.4 SST variability with 95% confidence interval shaded and, (**f**) simulated strength of the Pacific Walker circulation in the Nino4 region with 95% confidence interval shaded, according to the CSIRO Mk3L climate system model (see methods); (**g**) Standard deviation in mean annual rainfall record from Swallow Lagoon (as per Fig. 2c); eastern tropical Pacific measures of ENSO variability derived from (**h**) variance of individual foraminifera^11^ (grey bars; original sample at 7 cal kyr BP not shown as it is considered spurious by the authors), (**i**) bivalves^63^ (blue boxes), and (**j**) Laguna Pallcacocha variance, in 100-year non-overlapping windows, derived from normal-transformed data^64^ (black solid line). Inverted triangles represent the location of radiocarbon ages in the Swallow Lagoon record. LIA: Little Ice Age.

The nature of Holocene ENSO variance remains a subject of debate; central Pacific coral records suggesting no change in variance^9^ contrast with eastern Pacific ENSO proxies indicating enhanced variance in the late Holocene^11,19,25^. A recent analysis of coral and mollusc δ^18^O records from across the Pacific concluded marked changes in variance are in fact evident between the middle and late Holocene, and that tropical Pacific climate was susceptible to millennial-scale quiescent periods unrelated to orbital forcing^20^. In contrast, model simulations suggest the discrepancies between variability in eastern and central Pacific palaeoclimate data may be due to a differential response to insolation changes driven by orbital forcing^22^. Our record provides a new perspective from the southwestern Pacific and clearly demonstrate marked changes in rainfall variability over the last ~7700 years (Fig. 2). Prior to 5 cal kyr BP, variability is low, before a gradual increase ~5–2.5 cal kyr BP, and a further increase from ~1.2 cal kyr BP to present. The similarities between the timing of onset and trends in the variance between the eastern and western Pacific SSTs and teleconnected precipitation (Fig. 3) imply a common forcing mechanism.

Model simulations at 6 kyr and 0 kyr identify a strengthened Pacific Walker Circulation (PWC), driven by high boreal summer insolation and a stronger monsoon system, as the primary driver of reduced ENSO variability evident in proxy records during the mid-Holocene^7,26,27^. In this setting, strengthened trade winds foster more La Niña-like conditions and restrict the formation of El Niño events. To investigate the evolution of this scenario over time, we expand on these simulations using nine equilibrium climate model simulations spanning 8 kyr to 0 kyr, and derive metrics for the amplitude of ENSO variability and strength of the PWC (methods). Each simulation consists of a 1200-year model simulation (with the last 1000 years being used for analysis) and differs only via changes in the Earth’s orbital parameters. The model reproduces the long-term trends in ENSO variability over the last 8000 years seen in proxy records, with lower variability during the mid-Holocene (8–5 kyr) and gradually increasing late Holocene variability (Fig. 3). Modelled PWC strength suggests it reached a peak at 5 kyr, before decreasing towards 0 kyr. However, there is little difference between simulations either side of this peak, with the largest changes evident after 3 kyr, mirroring the pattern of rainfall variability at Swallow Lagoon (Fig. 3). Though it is likely that other factors beyond orbital forcing also influence ENSO, and the PWC, during the Holocene^28^, the simulations provide a mechanistic explanation for the coeval changes noted in proxy-ENSO records and rainfall in the Australian subtropics.

As our record tracks both wet and dry anomalies, we can characterise the shift at 3.2 cal kyr BP in terms of changes in the distribution as well as the amplitude of extremes. Prior to 3.2 cal kyr BP, no events exceeded ±2σ of the record mean; after 3.2 cal kyr BP, there are 12 dry excursions greater than 2σ, but only one wet excursion of this magnitude. While an increase in resolution towards the top of the record will naturally lead to the preservation of more short-lived events, we note that dry anomalies dominate and the transition towards an overall drier mean state, as illustrated by the GAM-LS (Fig. 2b), remains evident when the data are interpolated to a common centennial scale (Fig. S6). This trend suggests that the enhanced amplitude of late Holocene variability evident at Swallow Lagoon, and in other equatorial Pacific palaeoclimate records^19–21,25^, is driven by increasing strength of the El Niño phase alone, rather than simply a more variable system. While this has previously been implied from a marked shift in vegetation across eastern Australia towards more drought tolerant species around 3 cal kyr BP^29^, the Swallow Lagoon record confirms the one-sided nature of late-Holocene ENSO intensification.

The mid-Holocene (~7.7–3.5 cal kyr BP) at Swallow Lagoon is dominated by precipitation estimates above the mean (analogous to La Niña conditions in the instrumental record) though some dry periods are evident. The most extensive of these are apparent around 6.9, 6.8 and 5.8 cal kyr BP, suggesting El Niño was still active at this time. During the period 5.5–3.5 cal kyr BP, rainfall at Swallow Lagoon was generally stable, around a wet La Niña-like mean state. This period closely corresponds with a time of low variance in eastern Pacific SSTs^11^ from ~6–4 cal kyr and when Galápagos lake sediments suggest both phases of ENSO were less frequent^21^. Though some temporal smoothing is expected in the Galápagos record, as well as at Swallow Lagoon, the timing is also in general agreement with a “quiescent period” evident in high-resolution carbonate δ^18^O records from discrete periods between 5–3 cal kyr BP^20^. The GAM-LS model illustrates a period of very high rainfall at Swallow Lagoon around 3.5–3.0 cal kyr BP, which corresponds with a marked cool and dry period reflected in Galápagos lake sediments, also at 3.5–3.0 cal kyr BP^21^. Taken together, these findings suggest a centennial-scale period of enhanced zonal SST gradient, a persistently strong PWC and a more La Niña-like mean state.

The shift towards drier climates at Swallow Lagoon aligns with increasing SST variability in the eastern equatorial Pacific^11^ and the onset of more frequent El Niño events evident in sediment records from the Galápagos^19^ and Ecuador^25^ (Fig. 3; although we note the veracity of the Ecuadorean record in documenting El Niño events has recently been challenged^30^). Enhanced El Niño conditions in the west Pacific warm pool are evident in discreet coral records from Papua New Guinea around this time^31^, with notable prolonged and extreme events at ~2.5 and 2.04 ka corresponding with dry periods that exceed 2σ of the Swallow Lagoon record around 2.47 and 2.04 cal kyr BP. These events at Swallow Lagoon occur in a cluster of dry events during the ~2.6–2.0 cal kyr BP period, suggesting prolonged or extreme El Niño events, such as those evident in the coral records, may have occurred more regularly during this time. An absence of long coral records from the west Pacific precludes precise correlation with subsequent late-Holocene dry extremes in the Swallow Lagoon record, though a general agreement between rainfall trends (as illustrated by the GAM-LS) and west Pacific warm pool SSTs^32^ is evident through this period (Fig. 3).

A notable exception to the drier and more variable climate in the late Holocene at Swallow Lagoon is the stable high rainfall phase during the Little Ice Age (LIA: ~1450–1850)^8^, a period of globally cool temperatures^14,33^. ENSO variability during the LIA has been debated in recent research^8,14,34–36^. Problems in interpretation arise because of the heterogeneous relationship among terrestrial hydroclimate proxies, oceanic SST proxies and theoretical and physical models of predicted responses to globally cool periods^36–38^. A strengthened zonal gradient is indicated by hydrological records of a generally dry eastern Pacific^19,21,25^ contrasting with a wet western Pacific^34,36^, whereas a weakened zonal gradient is indicated by proxy records of relatively cool eastern and western Pacific SSTs^8,39^. The Swallow Lagoon record indicates persistently high rainfall during the LIA (Fig. 3). This is consistent with lake^40^ and tree-ring^41^ records from southern Australia that also find wet and low-variability LIA climate, which is inconsistent with El Niño-like conditions^14^. However, dry climate in northern Australia during the LIA^37^ is inconsistent with La Niña-like conditions. Thompson *et al*. (ref.^21^) suggest the pattern of reduced SST gradient described above is reminiscent of El Niño Modoki conditions; these can drive large-scale decreases in precipitation over northern Australia^42^, although they are unable to explain a wet southeastern^40^ or subtropical Australia (Swallow Lagoon). Given the critical impacts ENSO has on water resources in teleconnected regions, understanding this apparent disparity between SST and hydroclimate proxies highlights the need for further research into the response of ENSO to changes in global climate.

## Conclusions

Understanding ENSO variability is critically important because of its effects on precipitation regimes in teleconnected regions. The Swallow Lagoon precipitation record provides a new, quantitative, southwestern Pacific perspective of the influence of both ENSO phases over the mid- to late Holocene. The record has enabled, for the first time, an assessment of centennial- to millennial-scale variability in Australian subtropical rainfall. The pattern of low variability during the mid-Holocene, increasing after *ca*. 3 kyr cal BP, mirrors the variance evident in ENSO records from across the Pacific. The ~7.7–3.5 kyr cal BP period of low variability is characterised by predominantly wet climates at Swallow Lagoon, which implies a dominance of the La Niña phase during this time. After ~3 kyr cal BP, increasing variability is driven by the occurrence of extreme dry events, highlighting a strengthened El Niño phase as the primary driver of this change. Our climate model simulations implicate a progressive orbitally-driven weakening of Pacific Walker Circulation, particularly after 3 ka, as a contributing factor. At centennial scales, the record presents the first insights into subtropical Australian hydroclimates during the LIA, and we find that persistently high rainfall marks this period as anomalous in the context of the late Holocene. This contributes to a complex picture in which there is an apparent decoupling of SST and terrestrial hydroclimates during this interval. This requires further investigation as understanding ENSO response to radiative forcing is key to understanding the sensitivity of the system to anthropogenic climate changes^35^.

## Methods

### Chronology

An age model was developed based on 18 accelerator mass spectrometry (AMS) radiocarbon dates on short-lived terrestrial macrofossils (Table S1, Fig. S2). The samples were treated using the standard acid-alkali-acid pre-treatment to remove all carbon contamination. A radiocarbon chronology (Fig. S2) was constructed using the (Bayesian) OxCal P_Sequence deposition model with a low *k* parameter of 0.5 cm^−1^. The agreement index of the model A_model_ of 76% was good as it is higher than the accepted level of 60%^43^. Radiocarbon calibration data used for calendar age conversion are the post-bomb ^14^C data for Southern Hemisphere zone 1–2^44^ extended back in time by the SHCal13 calibration curve^45^. All calibrated ages are reported in cal. yr BP, with 0 yr BP being 1950 CE.

### Isotope analysis

All leaf samples were freeze dried for 24 hours and ground to a homogenous fine powder. Carbon isotope analyses were performed at the Natural Environment Research Council’s Isotope Geosciences Laboratory at the British Geological Survey in Nottingham, United Kingdom, by combustion in a Costech Elemental Analyser on-line to a VG TripleTrap and Optima dual-inlet mass spectrometer. δ^13^C values were calculated relative to the Vienna Pee-Dee Belemnite (VPDB) scale using within-run laboratory standards calibrated against NBS-18, NBS-19 and NBS-22. Replicate analysis of well-mixed samples indicated a precision of < 0.1‰ (1 SD).

### Precipitation reconstruction

There is a significant relationship (*r*^2^ = 0.64) between annual mean *M. quinquenervia* Δ_leaf_ *sensu* Farquhar *et al*. (ref.^46^) and mean annual rainfall^17^ which is improved slightly by taking into account the effect of atmospheric CO_2_ changes on Δ_leaf_. Δ_leaf_ in the Swallow Lagoon record was calculated using the atmospheric δ^13^C_atm_ from Elsig *et al*. (ref.^47^) between 7520 cal yr BP and 550CE and δ^13^C_atm_ for the remaining period calculated using Ferrio *et al*. (ref.^48^). We inferred rainfall using the relationship between rainfall and a discrimination anomaly^17^, that is, the difference between Δ_leaf_ predicted using Farquhar *et al*. (ref.^46^) and that predicted from CO_2_ using Schubert and Jahran (ref.^49^). We utilised CO_2_ data from Monin *et al*. (ref.^50^) for the period to 245 cal yr BP, from Law Dome^51^ from 245 to −20 cal yr BP (1970 CE) and from Cape Grim (www.csiro.au) from 1971 CE to present.

### Assessing variability

To simultaneously estimate trends in the mean and variance of the reconstructed rainfall time series requires a modelling approach that allows for linear predictors for each parameter of the conditional distribution of the response. Therefore, we chose to analyse the time series using a location scale generalized additive model (GAM-LS). Models of this type are also contained in the generalised additive model of location, scale, shape (GAMLSS) class^52^ and are more generally known as distributional models (e.g., ref.^53^). Our GAM-LS model includes smooth functions of time to simultaneously estimate trends in mean and variance of the observed time series. Because the models use smooth functions to estimate trends they do not require the response variable to be regularly spaced in time^54,55^. Furthermore, they do not suffer from edge effects in the same way as moving window methods, thus allowing continuous estimates of changes in variance over the entire time series. Edge effects do lead to increased uncertainty in trend estimates from GAM-LS models, but this additional uncertainty is accounted for in the standard errors of estimates from the model which are used to produce credible intervals for the estimated trends.

Rainfall values per year are typically observations of continuous random variables, bounded at 0. However, given the large rainfall values observed here, the Gaussian distribution is a close approximation for their conditional distribution. The Gaussian distribution is defined by two parameters; the mean (μ) and the standard deviation (σ). The GAM-LS approach allows both parameters to be modelled via separate linear predictors to capture variation in both the mean and the variance of the time series. The specific GAM-LS fitted here has the following form:

$$\begin{array}{rcl}{y}_{i} & \sim  & N({\mu }_{i},{\sigma }_{i}^{2})\\ {\mu }_{i} & = & \alpha +{f}_{1}(tim{e}_{i})\\ {log}({\sigma }_{i}-b) & = & \gamma +\beta t{i}_{i}+{f}_{2}(tim{e}_{i})\end{array}$$

which states that the *i*th δ^13^C observation (*y*_*i*_) is distributed Gaussian with mean *µ*_*i*_ and variance ${\sigma }_{i}^{2}$. We model *µ*_*i*_ as a smooth function (*f*_1_) of time (calibrated radiocarbon years BP) plus a constant term α (the model intercept). The linear predictor for *σ*_*i*_ is also modelled as a constant term, *γ*, plus a smooth function of time (*f*_2_), plus a linear parametric effect of the amount of time represented by each sample (*ti*_*i*_). *σ*_*i*_ is modelled on the log scale with a small lower bound *b* (0.01) to ensure parameter estimates remain positive and avoid issues with singularities in the likelihood of the model^56^. Any deviation from the assumed Gaussian distribution was assessed using standard model diagnostic plots for GAMs.

Thin plate spline bases were used for both smooth functions *f*_1_ and *f*_2_ with 200 and 75 basis functions respectively, to allow for potentially complex fitted trends in mean and variance. A penalty on the second derivative of the fitted smooths was used to control the amount of wiggliness in the estimated functions. Smoothness parameters, used to balance the fit and complexity of the model, were estimated via penalized maximum likelihood^57^. This has the effect of slightly biasing downwards the estimated variance trend. Dropping *f*_2_ from the model allows a test to be performed for a trend in variance over and above that which we might expect to observe due to varying sedimentation rates and time averaging present in each sample. Additionally, AIC was used to select between these two models.

Periods of significant change in the estimated trend in *σ*_*i*_ were identified using the first derivatives of *f*_2_, calculated using the method of finite differences. Periods of significant change exist where the 95% confidence interval on the first derivative of the smooth does not include a value of zero slope.

To investigate whether the estimated trend in *σ*_*i*_ was the result of variation in sedimentation rates, time averaging, and the uneven spacing of samples in time (not already accounted for by the *ti*_*i*_ term in the model), we compared our estimated variance trend with those estimated from 1000 null models fitted to simulated time series. Simulated time series $({\tilde{y}}_{i})$ were generated at an annual time step according to ${\tilde{y}}_{i}=N({\hat{\mu }}_{i},{\sigma }^{2})$, where ${\hat{\mu }}_{i}$ is the predicted trend from the full GAM-LS model described above but with constant standard deviation σ, determined from the standard error of the residuals of the GAM-LS fitted without *f*_2_ (hence we account for the heterogeneity implied by the varying amounts of time averaging in each sample when selecting the value of σ). Each annual value was assigned to a cm sediment slice following the age-depth relationship of the observed sediment record, and averaged to provide a mean value of the response for each slice. This process approximates the time averaging of individual years in the observed record. We then fitted the same GAM-LS model to each simulated series, including the smooth *f*_2_; any trend in *σ*_*i*_ estimated by *f*_2_ would be spurious, the result of either stochastic variation or the time averaging process, because the data were initially simulated with constant variance. We then compared the observed trend in *σ*_*i*_ with those estimated from the simulated time series (Fig. S4), and reject the null hypothesis that the observed trend in *σ*_*i*_ is a data artefact if it is extreme relative to the trends in the simulated series which were generated with no trend in variance. The GAM-LS models were estimated using the *mgcv* package^57^ for R^58^.

### Climate modelling

Snapshot simulations of the state of the global climate at 8, 7, 6, 5, 4, 3, 2 and 1 kyr BP, conducted using the CSIRO Mk3L climate system model v1.1^59,60^, were used. A pre-industrial control simulation provides the state of the global climate at 0 kyr BP. The snapshot simulations took into account the effects of orbital forcing, with the model being driven with the appropriate values of the Earth’s orbital parameters^61^ for each epoch. Otherwise, each snapshot simulation was identical to the pre-industrial control, with an atmospheric CO_2_ concentration of 280 ppm and a solar constant of 1365 Wm^−2^. The snapshot simulations were initialised from the state of the pre-industrial control simulation at the end of model year 100. Each snapshot simulation was then integrated for 1,100 years. The first 100 years were regarded as a spin-up period, with the final 1,000 years being used to derive statistics. The simulations are described in further detail by Phipps and Brown (ref.^62^).

For this study, two metrics were derived: (i) the amplitude of variability in El Niño-Southern Oscillation (ENSO), and (ii) the strength of the Pacific Walker Circulation. The amplitude of ENSO variability was diagnosed by calculating the monthly-mean sea surface temperature (SST) for the Niño 3.4 region (170–120°W, 5°S–5°N). A 2–7 year bandpass filter was then applied to extract ENSO variability. The ENSO amplitude was derived by calculating the standard deviation of the bandpass-filtered SST. The strength of the Pacific Walker circulation was diagnosed by calculating the monthly-mean strength of the zonal wind at 850 hPa for the Niño 4 region (160°E–150°W, 5°S–5°N). For both metrics, a block bootstrap was used to derive the 95% confidence interval.

## Supplementary information

Supplementary Information

## Data Availability

Data relating to this study can be found at https://figshare.com/s/b4b5431fd9577afd95ef.
