# Author Correction: Holocene El Niño–Southern Oscillation variability reflected in subtropical Australian precipitation

**DOI:** 10.1038/s41598-021-86998-2

**Published:** 2021-04-01

**Authors:** C. Barr, J. Tibby, M. J. Leng, J. J. Tyler, A. C. G. Henderson, J. T. Overpeck, G. L. Simpson, J. E. Cole, S. J. Phipps, J. C. Marshall, G. B. McGregor, Q. Hua, F. H. McRobie

**Affiliations:** 1 Department of Geography, Environment and Population, The University of Adelaide, North Terrace, Adelaide, South Australia 5005 Australia; 2 Sprigg Geobiology Centre, The University of Adelaide, North Terrace, Adelaide, South Australia 5005 Australia; 3 School of Biosciences, Sutton Bonington Campus, University of Nottingham, Leicestershire, LE12 5RD UK; 4 Stable Isotope Facility, Centre for Environmental Geochemistry, British Geological Survey, Nottingham, NG12 5GG UK; 5 Department of Earth Sciences, The University of Adelaide, North Terrace, Adelaide, South Australia 5005 Australia; 6 School of Geography, Politics and Sociology, Newcastle University, Newcastle upon Tyne, NE1 7RU UK; 7 School for Environment & Sustainability, The University of Michigan, Ann Arbor, Michigan 48109 USA; 8 Institute of the Environmental Change and Society, University of Regina, Saskatchewan, S4S0A2 Canada; 9 Department of Earth and Environmental Science, The University of Michigan, Ann Arbor, Michigan 48109 USA; 10 Institute for Marine and Antarctic Studies, University of Tasmania, Hobart, Tasmania Australia; 11 Queensland Department of Environment and Science, Dutton Park, Queensland 4102 Australia; 12 Australian Nuclear Science and Technology Organisation, Locked Bag 2001, Kirrawee DC, New South Wales 2232 Australia; 13 School of Mathematics and Statistics, University of Western Australia, Crawley, WA 6009 Australia

Correction to: *Scientifc Reports* https://doi.org/10.1038/s41598-019-38626-3, published online 07 February 2019

The original version of this Article contained an error in Reference 32, which was incorrectly given as:

Stott, L., Poulsen, C., Lund, S. & Thunell, R. Super ENSO and Global Climate Oscillations at Millennial Time Scales. *Science* **297**, 222–226 (2002).

The correct reference is listed below:

Stott, L., Cannariato, K., Thunell, R., Huag, G.H., Koutavas, A. & Lund, S. Decline of surface temperature and salinity in the western tropical Pacific Ocean in the Holocene epoch. *Nature* **431**, 56–59 (2004).

The original Article has been corrected.

