# Supplementary material for: Holocene El Niño–Southern Oscillation variability reflected in subtropical Australian precipitation
Source: Sci Rep. 2019 Feb 7;9:1627. doi: 10.1038/s41598-019-38626-3 (PMC6367503; doi:10.1038/s41598-019-38626-3)
Supplement: Supplementary file 1 — Supplementary Information [file 41598_2019_38626_MOESM1_ESM.pdf]

Title: Holocene El Niño–Southern Oscillation variability reflected in subtropical Australian precipitation

Authors: C. Barr, J. Tibby, M.J. Leng, J. J. Tyler, A.C.G. Henderson, J.T. Overpeck, G.L. Simpson, J.E. Cole, S.J. Phipps, J.C. Marshall, G.B. McGregor, Q. Hua, F.H. McRobie

Supplementary Information

<u>Site description</u>

Swallow lagoon is located high in the dunes (~150 m a.s.l) of North Stradbroke Island. As a perched lake on a sand island it is effectively the surface expression of a small aquifer contained within the surrounding sand. At the time of coring, the lake was 5.7 metres deep. Bimonthly monitoring determined that it is thermally stratified for 10 months of the year; the resulting anoxia likely to contributes to the preservation of leaves in the sediment. With no stream inflow or outflow, lake (and aquifer) level is determined by the balance of precipitation and evaporation. As such, a strong relationship ($r^2 = 0.85$) exists between lake level, as measured by depth loggers installed onsite, and rainfall deficit (Fig. S1). Mean annual rainfall on North Stradbroke Island is 1637 mm. Precipitation data is available from the Australian Bureau of Meteorology for Point Lookout Bowls Club (station number 040175) commencing in 1947, and Point Lookout (station number 40209) from 1997. The two stations are approximately one kilometre apart. Mean annual rainfall was calculated from a composite record of the two locations, as was rainfall data presented in Fig. S1.

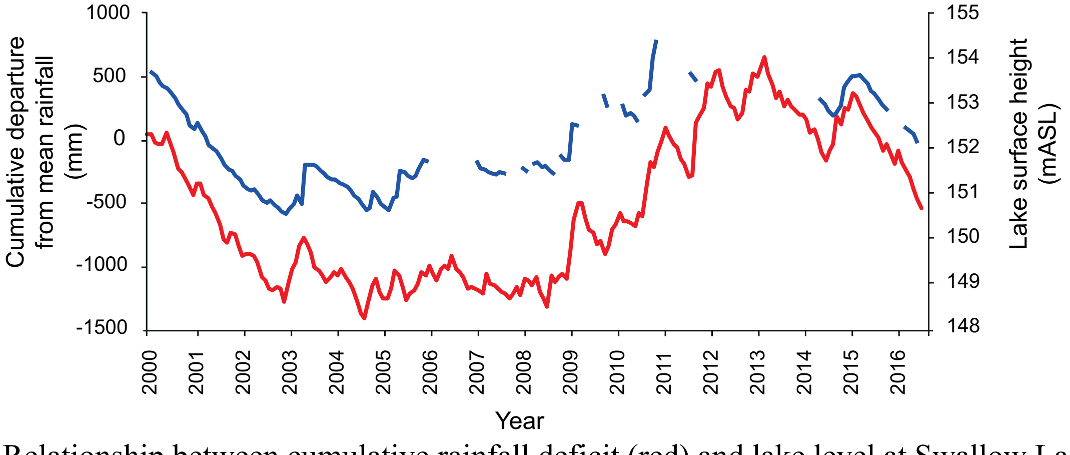

Fig. S1: Relationship between cumulative rainfall deficit (red) and lake level at Swallow Lagoon (blue).

Swallow Lagoon is fringed by a small community of *Melaleuca quinquenervia*, an evergreen tree native to Papua New Guinea, New Caledonia and the tropical and subtropical east coast of Australia where it fringes freshwater lakes and dominates intermittently inundated coastal floodplains. The time between leaf formation and leaf abscission in *M. quinquenervia* can vary from 2 to 4 years[1]. As the lake is geologically and hydrologically isolated from other lakes and wetlands on the island, and the next nearest *M. quinquenervia* stand is at Brown Lake approximately 2 km away, we are confident that the *M. quinquenervia* leaves picked from the sediment cores originate from this local community.

<u>Core collection</u>

The Swallow Lagoon record is a composite of three sediment cores collected from the deepest part of the lake. As the uppermost 150 cm of sediment had very high water content, soft-sediment push corers were used to maintain stratigraphic integrity of the sediment. To maximise collection of leaves and leaf fragments, a large-diameter soft-sediment core was taken of the top 94 cm (core SLP2). A second small-diameter core (core SLP3) was taken of the top 250 cm to ensure penetration into the firm underlying sediment. Both were extruded at 1 cm intervals in the field. Core SL-2 (150–370 cm) was collected using a Livingstone corer and was correlated to SLP-3 using a conspicuous 7 cm thick band of sand that was evident at a depth of 235 cm in each core.

Macrofossil sampling

The sediment was sampled at contiguous 1 cm-equivalent intervals after correction for compaction. Each sample was sieved and the >500 μm fraction was retained. *M. quinquenervia* leaf fragments were identified under stereoscope by obvious morphological features, such as thick veins, epidermal colour and parenchymal cells in the spongy mesophyll. The leaf fragments varied in size from several cm^2 to several mm^2. Leaves of other taxa (e.g., Eucalyptus) were occasionally present though were not used in analyses. Macrofossil samples for radiocarbon dating were also picked from the sieved remnants.

Precipitation reconstruction

Carbon isotope discrimination by C_3 plants relative to the atmosphere (Δ_{leaf})[2] is significantly related to rainfall at global[3] and regional[4] scales as moisture availability is the primary driver of leaf gas exchange in modern plants[3]. Recently, Schubert and Jahren (ref. 5) have argued that changes in atmospheric CO_2 can also influence Δ_{leaf}. In addition, the strength of the Δ_{leaf}-rainfall relationship is species-specific because of plant phylogeny and leaf traits. Other factors that can affect Δ_{leaf} are location-specific, such as canopy density, degree of shading and the hydrological stress of individual trees[3]. In this study, we can control for inter-species variation as leaves from only one tree species (*M. quinquenervia*) are analysed. Intra-species variation in *M. quinquenervia* are accounted for as the tree species only occurs within 4 metres of Swallow Lagoon and, as North Stradbroke Island is a sand island, constant soil type and consistent hydrological stressors exist within this local *Melaleuca* community. In addition, the broad open canopy precludes any transpiration effects on 'shade' leaves[6]. For these reasons, the species-specific calibration dataset of Tibby et al (ref. 7) has advantages over other global datasets for this study[3,8]

Assessing variability

Standard statistical methods that might be applied to these data typically focus on estimating the trend in the mean of the response, making the assumption that the variance or other moments are constant across observations. Visual inspection of the $\delta^{13}C$ calibrated rainfall time series suggested the presence of both a nonlinear trend and an increase in the variance of the observations. A trend in variance would violate any assumption of homogeneity. Furthermore, it is of significant interest to estimate the magnitude of any change in variance of the climate system as recorded at Swallow Lagoon.

Previous approaches for estimating change in variance in (palaeo)environmental time series have adopted a moving window approach (e.g. refs 9 and 10), in which the variance of a set of observations within a window of known width (typically ≤ half the time series length) is computed[11]. The window is shifted forward in time by one observation and the process repeated. Typically, the moving variance is computed using a detrended version of the time series.

There are a number of significant issues with the moving window approach: i) the irregular spacing of samples in time in a typical palaeoenvironmental time series is not easily accommodated and remedying the irregularity via interpolation can induce changes in the distributional properties of the data that we are interesting in estimating (e.g. ref. 12), ii) significance testing of the derived

moving variance time series is complex due to strong autocorrelation patterns introduced because a moving window contains all but one sample used in the estimation of the variance in the previous window, iii) it is not possible to simultaneously estimate the mean and variance of a time series using the moving window approach, and detrending the series prior to estimating the moving variance risks conflating changes in variance with changes in the mean, biasing estimates of both, and iv) a significant reduction in power to detect change in variance arises via edge effects because the window width used requires us to observe as many observations as are included in the window before the first estimate of the variance can be calculated (if using a right padded moving window) or observations equal to half the window width if using a centred window.

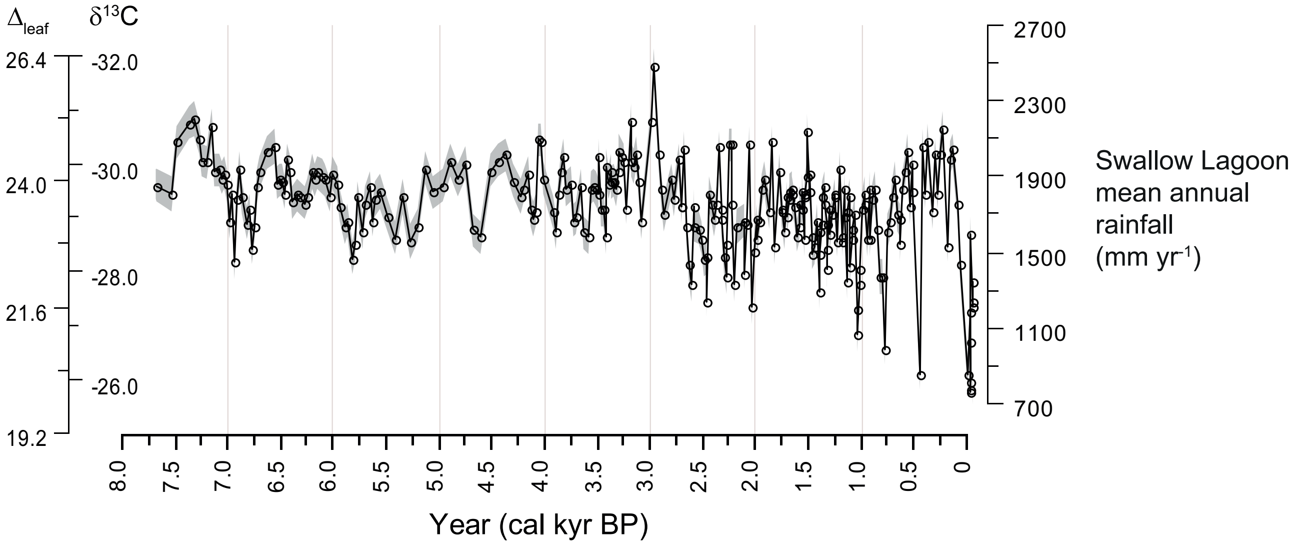

Fig. S2: $\delta^{13}C$ leaf, Δ_{leaf} and inferred mean annual rainfall from Swallow Lagoon. Grey shading represents the standard error (+/- 88 mm) for the rainfall reconstruction.

Table S1: Radiocarbon results and modelled cal. ages at 95% probability.
Samples with laboratory numbers commencing with "Beta-" were analysed by Beta Analytics, with "Wk-" by Waikato Radiocarbon Laboratory, and with "OZ" by the Australian Nuclear Science and Technology Organisation [13]
* - Post-bomb date; fraction of modern carbon ($F^{14}C$), instead of ^{14}C age, is reported.

Depth (cm)	Laboratory no.	Material dated	^{14}C Age (BP) ± 1σ	Modelled age (median, range; cal yr BP)
18	OZP517	Melaleuca leaves	1.2589 ± 0.0029*	–12; -32 to –11
33	Beta-382306	Melaleuca leaves	300 ± 30	376; 284–444
39	OZP518	Melaleuca leaves	560 ± 25	531; 509–551
57	OZP519	Melaleuca leaves	1050 ± 25	925; 819–960
63	OZO275	Melaleuca seed pod	1220 ± 30	1012; 969–1098
75	OZP520	Melaleuca leaves	1280 ± 25	1172; 1094–1264
86	Wk-31579	Melaleuca leaves	1520 ± 25	1335; 1307–1374
93	OZP521	Melaleuca leaves	1560 ± 25	1395; 1352–1452
116	OZO276	Leaves	1710 ± 30	1655; 1540–1702
160	OZO206	Seed Pod	2385 ± 30	2360; 2315–2483
212	OZO207	Twig	3205 ± 30	3389; 3273–3454
220	OZO208	Seed	3310 ± 30	3530; 3448–3586
231	OZO209	Seed Pod	3610 ± 30	3860; 3726–3965
244	OZO210	Twigs Bark	3820 ± 30	4203; 4084–4381
264	OZO277	Twigs Bark	4935 ± 30	5619; 5489–5707
290	OZO278	Leaves	5750 ± 30	6480; 6405–6602
314	Wk-31580	Melaleuca leaves	6210 ± 30	7104; 6976–7231
329	OZO279	Leaves and seed	6920 ± 30	7693; 7616–7783

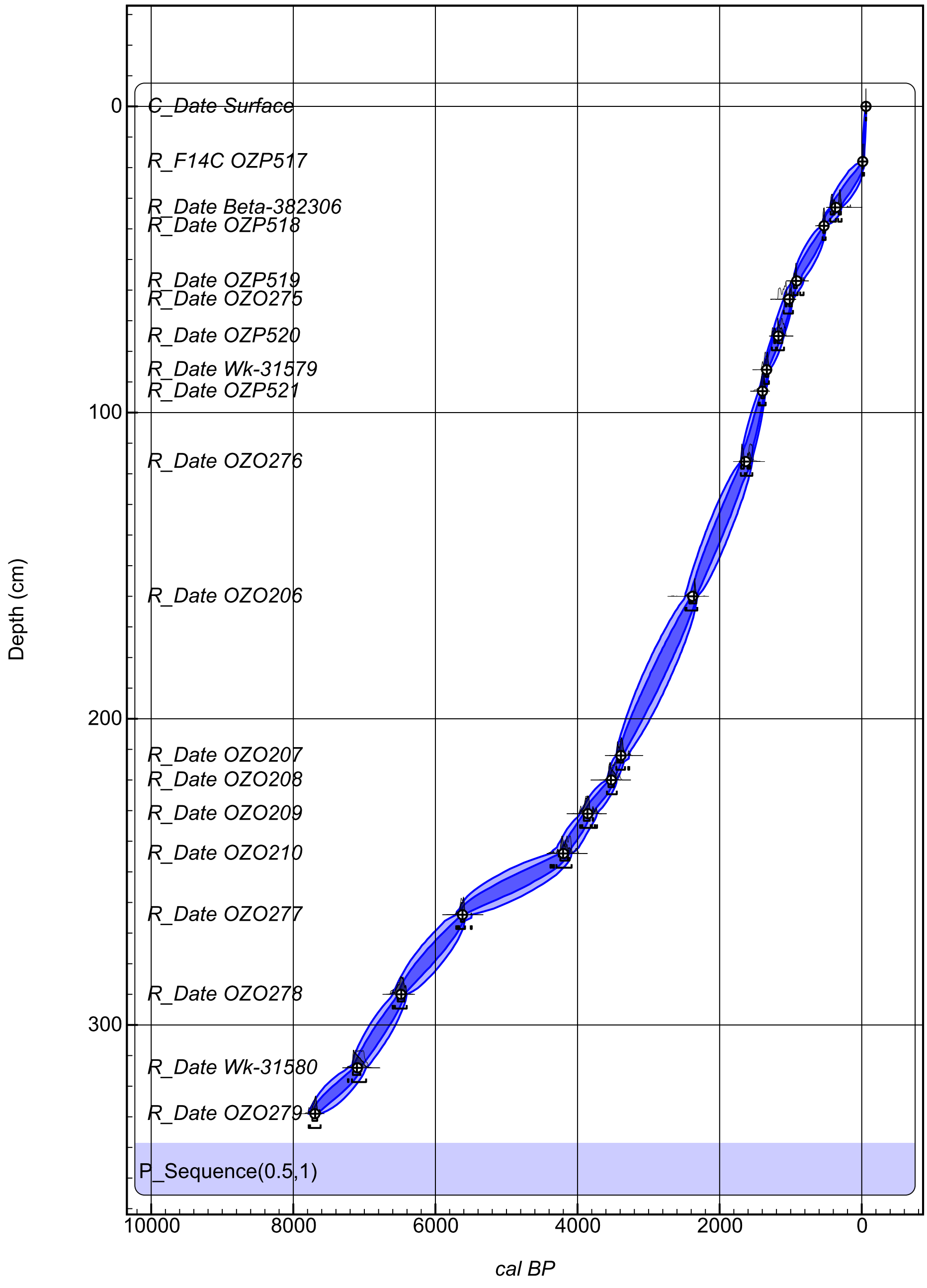

Fig. S3: Age-depth model for the Swallow Lagoon record

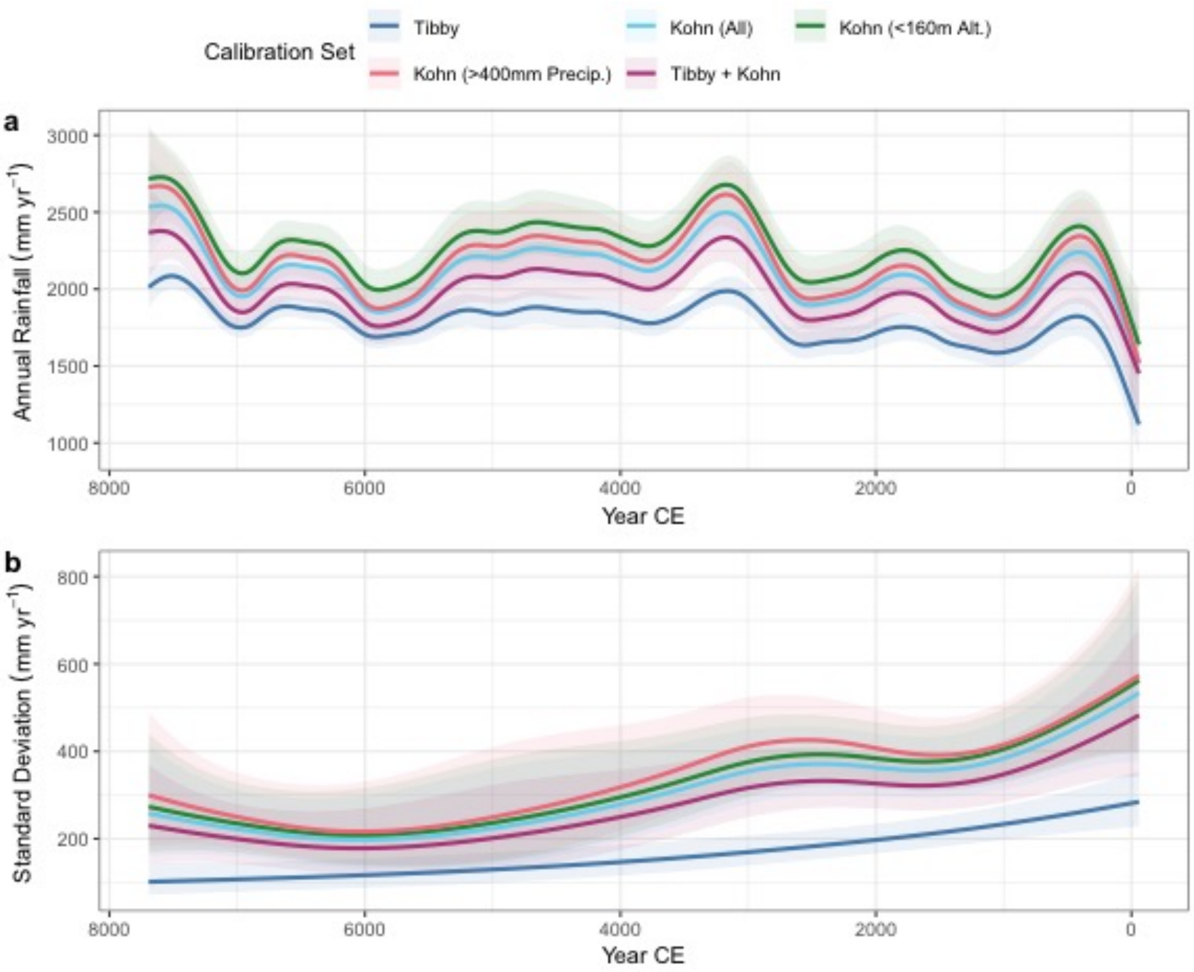

Fig S4: GAM-LS models (**a.**) and variance (**b.**) of rainfall reconstructions based on the differing calibration datasets of Tibby et al (ref. 7) and Kohn (ref. 8). Apart from using the entire Kohn dataset (Kohn (all)), we also examined subsets of this dataset that were relevant to the modern distribution of *Melaleuca quinquenervia*; that is, only sites below 160 m altitude, and only sites that had mean annual precipitation greater than 400 mm yr^{-1}. The 'Tibby+Kohn' calibration is the combination of 'Tibby' and 'Kohn (all)'. With the exception of the 'Tibby' dataset, which has a linear relationship, all other datasets used a 2nd order polynomial relationship. Comparisons of the reconstructions demonstrate that enhanced late Holocene rainfall variability is evident regardless of which calibration dataset is used. Note the different y-axis scale in b. compared to Fig S5.

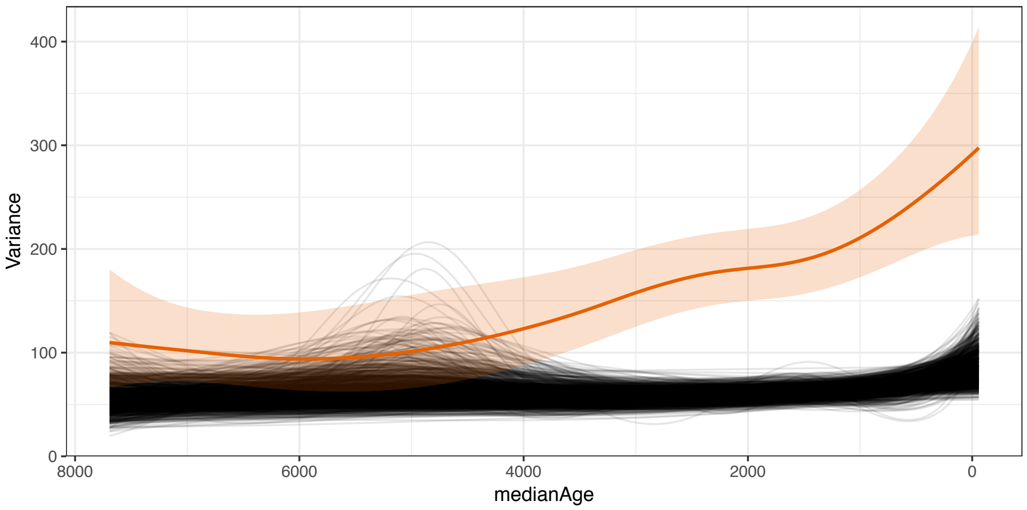

Fig. S5: Results of 1000 null models (thin black lines) compared to variance in the Swallow Lagoon rainfall record, demonstrating the trend in variance is not an artefact arising from varying sampling resolution in time.

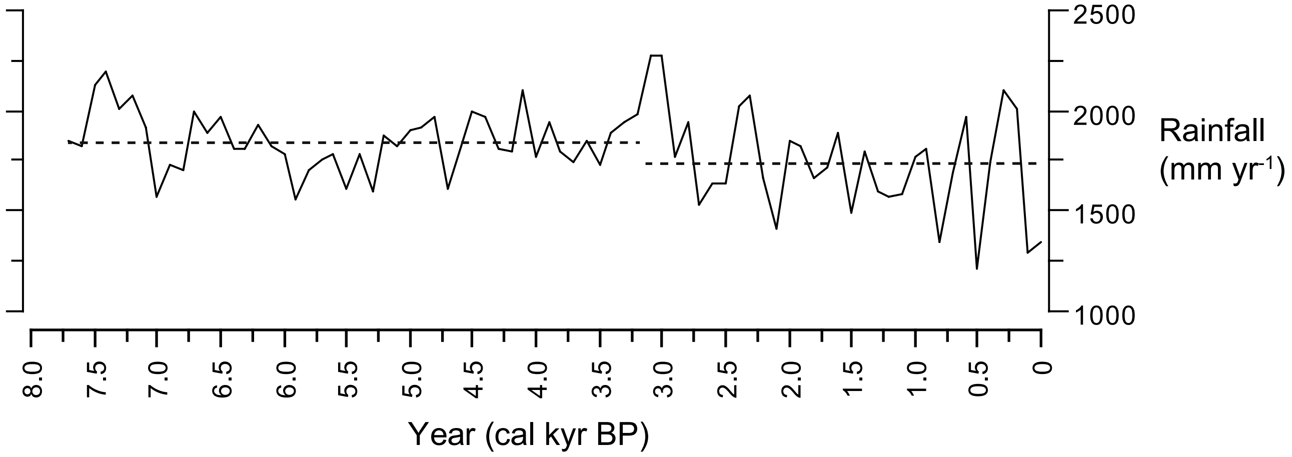

Fig. S6: Rainfall data interpolated to centennial-scale (the lowest temporal resolution of the record) illustrating a shift to drier conditions in the late-Holocene. Dashed lines represent mean inferred-rainfall prior to 3.2 cal kyr BP (1844 mm yr^{-1}, σ = 145 mm yr^{-1}) and after 3.2 cal kyr BP (1742 mm yr^{-1}, σ = 267 mm yr^{-1}).

REFERENCES

1 Van, T. K., Rayachhetry, M. B., Center, T. D. & Pratt, P. D. Litter dynamics and phenology of *Melaleuca quinquenervia* in south Florida. *Journal of Aquatic Plant Management* **40**, 22-27 (2002).

2 Farquhar, G. D., Ehleringer, J. R. & Hubick, K. T. Carbon isotope discrimination and photosynthesis. *Annual Review of Plant Physiology and Plant Molecular Biology* **40**, 503-537 (1989).

3 Diefendorf, A. F., Mueller, K. E., Wing, S. L., Koch, P. L. & Freeman, K. H. Global patterns in leaf ^{13}C discrimination and implications for studies of past and future climate. *Proceedings of the National Academy of Sciences* **107**, 5738-5743, doi:10.1073/pnas.0910513107 (2010).

4 Stewart, G. R., Turnbull, M. H., Schmidt, S. & Erskine, P. D. ^{13}C Natural abundance in plant communities along a rainfall gradient: a biological integrator of water availability. *Australian Journal of Plant Physiology* **22**, 51-55 (1995).

5 Schubert, B. A. & Jahren, A. H. The effect of atmospheric CO2 concentration on carbon isotope fractionation in C3 land plants. *Geochimica et Cosmochimica Acta* **96**, 29-43, doi:10.1016/j.gca.2012.08.003 (2012).

6 Lockheart, M. J., Poole, I., Van Bergen, P. F. & Evershed, R. P. Leaf carbon isotope compositions and stomatal characters: important considerations for palaeoclimate reconstructions. *Organic Geochemistry* **29**, 1003-1008 (1998).

7 Tibby, J. *et al.* Carbon isotope discrimination in leaves of the broad-leaved paperbark tree, *Melaleuca quinquenervia*, as a tool for quantifying past tropical and subtropical rainfall. *Global Change Biology* **22**, 3474–3486 (2016).

8 Kohn, M. J. Carbon isotope compositions of terrestrial C3 plants as indicators of (paleo)ecology and (paleo)climate. *Proceedings of the National Academy of Sciences of the United States of America* **107**, 19691-19695 (2010).

9 Dakos, V. *et al.* Slowing down as an early warning signal for abrupt climate change. *Proceedings of the National Academy of Sciences* **105**, 14308-14312 (2008).

10 Wang, R. *et al.* Flickering gives early warning signals of a critical transition to a eutrophic lake state. *Nature* **492**, 419-422 (2012).

11 Dakos, V. *et al.* Methods for detecting early warnings of critical transitions in time series illustrated using simulated ecological data. *PloS one* **7**, e41010 (2012).

12 Carstensen, J., Telford, R. J. & Birks, H. J. B. Diatom flickering prior to regime shift. *Nature* **498**, E11-E12 (2013).

13 Fink, D. *et al.* The ANTARES AMS facility at ANSTO. *Nuclear Instruments and Methods in Physics Research B* **223-224**, 109-115 (2004).
